# The Common Clinical Presentation of Patients Selected for Septoplasty in Northern Saudi Arabia

**DOI:** 10.1155/2018/8536387

**Published:** 2018-06-03

**Authors:** Abdullah D. Alotaibi, Bassam Ahmed Almutlaq, Fahad Nashmi Alshammari, Hussain Gadelkarim Ahmed

**Affiliations:** ^1^University of Hail, College of Medicine, Department of Otolaryngology Head and Neck Surgery, Hail, Saudi Arabia; ^2^University of Hail, College of Medicine, Hail, Saudi Arabia; ^3^King Khalid Hospital, Department of Otolaryngology Head and Neck Surgery, Hail, Saudi Arabia

## Abstract

**Background:**

Nasal septal deviation (NSD) plays a critical role in nasal obstruction symptoms, aesthetic look of the nose, increased nasal resistance, and occasionally snoring. Septoplasty is the most common method for correction of deviated nasal septum (DNS). Therefore, the aim of the present study was to assess the association between initial clinical presentations of patients selected for septoplasty and demographical characteristics in Northern Saudi Arabia.

**Methodology:**

Archives related to all patients selected for septoplasty between 2012 and 2017 were retrieved from ENT Department at King Khalid Hospital in Hail, Northern Saudi Arabia. Only adults over 18 years of age were included in this study.

**Results:**

With regard to the clinical presentations, almost all patients presented with variable degrees of nasal congestions, nasal blockages, breathing troubles, sleeping troubles, and exercise problems.

**Conclusion:**

Nasal obstruction is prevalent in Northern Saudi Arabia with peaks being in the years 2016 and 2014 with the most etiological factor being DNS.

## 1. Introduction

Surgical and medical management of nasal obstruction is the common measure in otolaryngologist practice. The perfect management of deviated nasal septum (DNS) is septoplasty [1]. Chronic nasal airway obstruction is one of the most frequent complaints that patients complain of to otolaryngologists. DNS is a common anatomic disparity and the most common cause of nasal obstruction [2–4]. Surgical amendment of the DNS is the most frequent ear, nose, and throat (ENT) operation in adults [5]. 

Septoplasty is a surgical procedure that is done to correct a DNS. A DNS arises when the cartilage that separates the nostrils is out of place. This can cause various breathing difficulties. Septoplasty is one of the most frequently performed otorhinolaryngological procedures, which might be very challenging for many surgeons. A precise preoperative diagnosis of pathologies of the septum in the situation of the nasal cavity is crucial for the success of surgery. Intraoperative visualization through microscope or endoscope is very supportive for the surgeon and for the training of the residents. The new method of septoplasty with the phases of methodology, mobilization, resection/repositioning, and reconstruction/fixation is offered. As particularly pathologies of the caudal septum are responsible for failures of septal surgery, some special difficulties of this region such as the vertical fracture of the caudal septum, the deficiency of caudal septum, or anterior convexities of the cartilaginous septum are debated [6, 7].

Annual septoplasty rates, however, vary between countries. More than 20,000 septoplasties, (3.8 septoplasties per 10,000 inhabitants) were done in England between 2012 and 2013 [8]. In spite of the significant number of septoplasties achieved each year, complications after this process are relatively rare. Most complications result from insufficient surgical preparation or poor performance and often can be avoided. Surgeons should discuss these risks with patients before surgery as part of the informed consent process [9].

Patients selected for septoplasty present with diverse clinical presentations, particularly those with DNS. The occurrence of these clinical presentations may differ in association with demographical features of patients in specific population. Therefore, the aim of the present study was to assess the association between initial clinical presentations of patients selected for septoplasty and demographical characteristics in Northern Saudi Arabia.

## 2. Materials and Methods

This study involved 131 patients that presented with nasal obstruction and that were subsequently selected for septoplasty. Archives related to all selected patients for septoplasty between 2012 and 2017 were retrieved from ENT Department at King Khalid Hospital in Hail, Northern Saudi Arabia. Only adults over 18 years of age were included in this study. Patients' medical records were investigated, and patients with a history of rhinoplasty, cranial and facial trauma, or bone deformity (except DNS) and patients with mass in the nasal cavity were excluded from the study. Several nasal obstruction related clinical presentations were recorded, including nasal congestion, nasal blockages, breathing troubles, sleeping troubles, and exercise problems. Demographical characteristics including age, gender, and residence were also recorded.

Patients were assigned to septoplasty (with or without concurrent turbinate surgery) and the procedure was performed within 3 to 5 weeks after final surgical treatment choice.

## 3. Ethical Consent

Our study protocol conformed with the 2013 Declaration of Helsinki and this study was approved by the ethics committee of the College of Medicine, University of Hail, Saudi Arabia.

## 4. Statistical Analysis

Statistical analysis was performed using SPSS software for Windows (version 16.0, SPSS Inc., Chicago, IL, USA). Categorical variables are given as frequencies and percentages, as well as continuous variables. For all statistical comparisons, a *p* value below 0.05 was considered statistically significant.

## 5. Results

**Figure 1:**
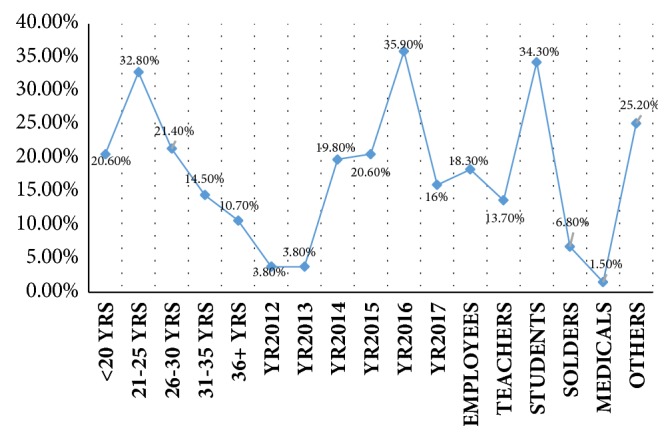
Description of the study population by age, year of admission, and occupations.

**Figure 2:**
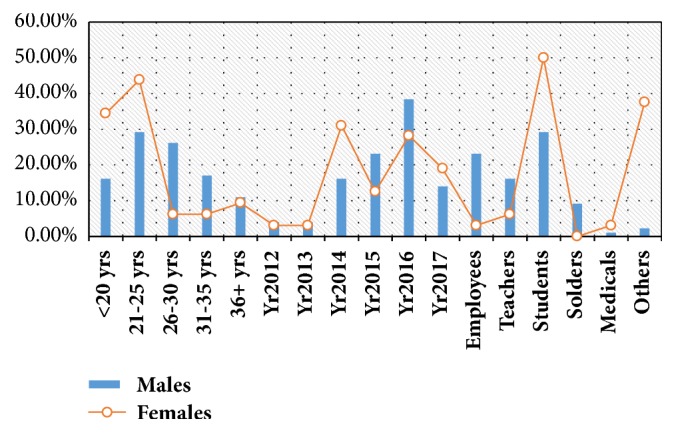
Description of the study population by gender and demographical characteristics.

The present study investigated retrospectively 131 consecutive cases of nasal blockages related abnormalities, their ages ranging from 18 to 42 years, with a mean age of 26 years. Most of the patients were 21–25 years old, followed by age ranges 36–30, <20 years, and 31–35 years, representing 43, 28, 27, and 19, respectively, as indicated in Table 1 and Figure 1. The majority of males were found at age group 21–25 years followed by 26–30 and 31–35 years constituting 29, 26, and 17, respectively. The majority of females were found at age group 21–25 years, followed by <20 years constituting 14 and 11 correspondingly, as indicated in Table 1 and Figures 1 and 2.

Out of 131 patients, 99/131 (75.6%) were males and 32/131 (24.4%) were females, giving male-to-female ratio of 3.09 to 1.00. The majority of the patients were registered in the year 2016 followed by years 2015, 2014, 2017, 2012, and 2013, representing 47 (35.9%), 27 (20.6%), 26 (19.8%), 21 (16.1%), and 5 (3.8%), respectively. For males, most of them presented in the year 2016 followed by years 2015, 2014, 2017, 2013, and 2012, constituting 38/99 (38.4%), 23/99 (23.2%), 16/99 (16.2%), 14/99 (14%), and 4/99 (4.1%), respectively. For females, most of them presented in the year 2014 followed by years 2016 and 2017, representing 10/32 (31%), 9/32 (28.2%), and 7/32 (19%), in this order, as shown in Table 1 and Figures 1 and 2.

With regard to the occupations, the majority of the patients were students representing 45/131 (34.4%) followed by others, employees, teachers, and soldiers, constituting 33 (25.2%), 24 (18.3%), 18 (13.7%), and 9 (6.8%), in this order. For males, the great majority were students followed by employees and others representing 29/99 (29.3%), 23 (23.2%), and 21 (21.2%), correspondingly. For females, the majority were students followed by others, constituting 16/32 (50%) and 12 (37.5%), respectively, as indicated in Table 1 and Figures 1 and 2.

**Figure 3:**
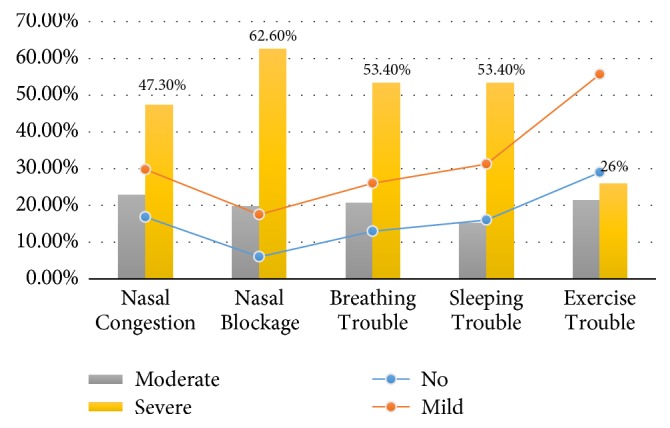
Description of patients by initial clinical presentation.

With regard to the clinical presentations, almost all patients presented with variable degrees of nasal congestions, nasal blockages, breathing troubles, sleeping troubles, and exercise problems. For nasal congestions, 17 (13%), 30 (22.9%), and 62 (47.3%) of the patients presented with mild, moderate, and severe nasal congestions, respectively. For nasal blockages, 15 (11.5%), 26 (19.8%), and 82 (62.6%) of the patients presented with mild, moderate, and severe nasal blockages. For breathing troubles, 17 (13%), 27 (20.6%), and 70 (53.4%) of the patients presented with mild, moderate, and severe breathing trouble. For sleeping troubles, 20 (15.3%), 20 (15.3%), and 70 (53.4%) of the patients presented with mild, moderate, and severe sleeping troubles. For exercise troubles, 31 (26.7%), 28 (21.3%), and 34 (26%) of the patients presented with mild, moderate, and severe exercise troubles, as indicated in Table 2 and Figure 3.

**Figure 4:**
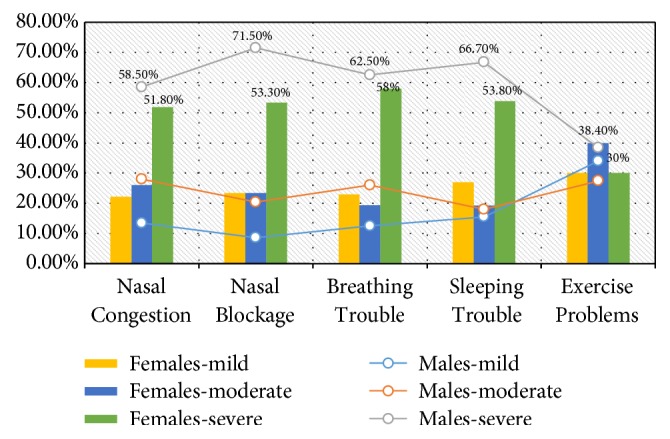
Description of patients by initial clinical presentations and gender.

Table 3 summarizes the distribution of the study subjects by gender and clinical presentations. Out of 82 male patients that presented with nasal congestions, 11/82 (13.4%), 23 (28%), and 48 (58.5%) of the patients presented with mild, moderate, and severe nasal congestions, respectively, and the relative risk (RR) (95% confidence interval, 95% CI) was 0.98 (0.8311 to 1.1736). Out of 27 female patients that presented with nasal congestions, 6/27 (22.2%), 7 (26%), and 14 (51.8%) of the patients presented with mild, moderate, and severe nasal congestions, respectively, as indicated in Table 3 and Figure 4.

Out of 93 male patients that presented with nasal blockages, 8/93 (8.6%), 19 (20.4%), and 66 (71%) of the patients presented with mild, moderate, and severe nasal blockages, respectively, and the RR (95% CI) was 0.96 (0.2058 to 4.5683). Out of 30 female patients that presented with nasal blockages, 7/30 (23.3%), 7 (23.3%), and 16 (53.3%) of the patients presented with mild, moderate, and severe nasal blockages, respectively, as indicated in Table 3 and Figure 4.

Out of 88 male patients that presented with breathing troubles, 11/88 (12.5%), 22 (26%), and 55 (62.5%) of the patients presented with mild, moderate, and severe breathing troubles, respectively, and the RR (95% CI) was 1.09 (0.9148 to 1.3116). Out of 26 female patients that presented with breathing troubles, 6/26 (23%), 5 (19.2%), and 15 (58%) of the patients presented with mild, moderate, and severe breathing troubles, respectively, as indicated in Table 3 and Figure 4.

Out of 84 male patients that presented with sleeping troubles, 13/84 (15.5%), 15 (18%), and 56 (66.7%) of the patients presented with mild, moderate, and severe sleeping troubles, respectively, and the RR (95% CI) was 1.04 (0.8670 to 1.2579). Out of 26 female patients that presented with sleeping trouble, 7/26 (27%), 5 (19.2%), and 14 (53.8%) presented with mild, moderate, and severe sleeping troubles, respectively, as indicated in Table 3 and Figure 4.

Out of 73 male patients that presented with exercise problems, 25/73 (34%), 20 (27.4%), and 28 (38.4%) of the patients presented with mild, moderate, and severe exercise problems, respectively, and the RR (95% CI) was 1.17 (0.8802 to 1.5815). Out of 20 female patients that presented with sleeping troubles, 6/20 (30%), 8 (40%), and 6 (30%) of the patients presented with mild, moderate, and severe exercise problem, respectively, as indicated in Table 3 and Figure 4.

The distribution of the study subjects by age and clinical presentations is summarized in Table 4. Most of the cases with severe nasal congestions were found among age group 26–30 years followed by age range 21–25 representing 17/62 (27.4%) and 16/62 (25.8%), respectively; hence, the cases with moderate degree were found among age group 21–25 years followed by age group <20 years constituting 14/30 (46.7%) and 5/30 (16.7%), in this order.

Most of the cases with severe nasal blockages were found among age group 21–25 years followed by age group <20 representing 24/62 (38.7%) and 16/62 (25.8%), respectively; hence, most of the cases with moderate degree were found among age group <20 years followed by age group 21–25 years constituting 9/30 (30%) and 8/30 (26.7%), in this order, as indicated in Table 4.

Most of the cases with severe breathing troubles were found among age group 21–25 years followed by age group 26–30 representing 24/62 (38.7%) and 18/62 (29%), respectively; hence, most of the cases with moderate degree were found among age group 21–25 years followed by <20 years constituting 7/30 (23.3%) and 3/30 (10%), in this order, as indicated in Table 4.

Most of the cases with severe sleeping troubles were found among age group 21–25 years followed by age group 26–30 representing 19/62 (30.6%) and 15/62 (24.2%), respectively; hence, most of the cases with moderate degree were found among age group 21–25 years followed by 26–30 years constituting 7/30 (23.3%) and 6/30 (20%), in this order, as indicated in Table 4.

Most of the cases with severe exercise problems were found among age group 21–25 years followed by 26–30 representing 13/62 (21%) and 7/62 (11.3%), respectively; hence, most of the cases with moderate degree were found among age group 21–25 years followed by 26–30 years constituting 12/30 (40%) and 6/30 (20%), in this order, as indicated in Table 4.

The distribution of the study subjects by occupations and clinical presentations is summarized in Table 4. The majority of patients with severe nasal congestions were found among students followed by others constituting 19/62 (30.6%) and 16 (25.8%) correspondingly, whereas the majority of cases with moderate condition were identified among students and employees representing 9/30 (30%) and 8 (26.7%), respectively.

The majority of patients with severe nasal blockages were found among students followed by others constituting 29/62 (46.8%) and 19 (30.6%) correspondingly, whereas the majority of cases with moderate conditions were identified among students and employees representing 11/30 (36.7%) and 6 (20%), respectively, as indicated in Table 5.

The majority of patients with severe breathing troubles were found among students followed by others constituting 25/62 (40.3%) and 17 (27.4%) correspondingly, whereas the majority of cases with moderate conditions were identified among students and employees representing 10/30 (33.3%) and 6 (20%), respectively, as indicated in Table 5.

The majority of patients with severe sleeping troubles were found among students followed by employees constituting 25/62 (40.3%) and 16 (%) correspondingly, whereas the majority of cases with moderate conditions were identified among students and employees representing 7/30 (23.3%) and 5 (16.7%), respectively, as indicated in Table 5.

The majority of patients with severe exercise problems were found among students followed by employees constituting 25/62 (40.3%) and 16 (25.8%) correspondingly, whereas the majority of cases with moderate conditions were identified among students and others representing 8/30 (26.7%) and 8 (26.7%), respectively, as indicated in Table 5.

Out of 131 patients, 112 (85.5%) were diagnosed with DNS and the remaining 19 (14.5%) were found with conditions other than DNS. All of the patients were selected for septoplasty. Out of 99 males, 87 (87.9%) were found with DNS; hence, out of 32 females, 25 (78%) were found with DNS.

## 6. Discussion

Nasal obstruction is a common problem, which is caused by several factors with the greatest being DNS. The nasal septum has a vital role in both the look and function of the nose. Deviation of the nose is reciprocal and amendment needs an attentive, anatomically based treatment. Therefore, the present study focused on the association between initial clinical presentation (mainly nasal obstruction) of patients selected for septoplasty and demographical characteristics in Northern Saudi Arabia.

In the present study, out of 131 patients with nasal obstruction, about 76% were males and only 24% were females. DNS is a very prevalent condition in several parts of the world, with severe symptoms such as nasal obstruction and rhinitis. Although some studies have showed similar results to our findings that DNS is more prevalent in men [10, 11], some epidemiology studies reported that women have nearly double the rate of chronic rhinosinusitis (CRS) when compared with men [12, 13], whereas other studies found no difference [11, 14]. Studies from Saudi Arabia have shown that DNS is more common among males than females [15].

In the present study, DNS was more common in age range 21 to 29 years. Although diverse studies reported varying percentages with mostly age mean around 30 years old, similar results have been previously reported [16, 17].

The great majority of the cases in this series were reported in the year 2016 followed by year 2014. Although in our extensive search regarding the epidemiology of DNS we did not come across a study that compares the prevalence of DNA in different years, singly scattered reports from Saudi Arabia were relatively comparable to our findings [17, 18]. Annual septoplasty rates, however, differ between countries. More than 20,000 septoplasties, i.e., 3.8 septoplasties per 10,000 inhabitants, were performed in England between 2012 and 2013[8]. In the Netherlands, 10,000 septoplasties, i.e., 6.0 septoplasties per 10,000 inhabitants, were performed as a single procedure or in combination with turbinate surgery in 2010 [19].

With regard to occupations, most of the patients were students followed by employees. However, not all of our study population were cases of DNS; some were cases of nasal obstruction due to other etiological factors. Reversible nasal congestion is usually caused by mucosal inflammation and secretions. In contrast, fixed or relatively constant congestion (i.e., obstruction) may be due to occlusion (e.g., nasal polyps, foreign body), anatomical variation (e.g., septal deformity, turbinate hypertrophy), or, rarely, neoplasm. In some cases, abnormal sensory perception may also contribute to a patient's perception of nasal congestion. A differential diagnosis of reversible nasal congestion includes allergic rhinitis, nonallergic rhinitis, vasomotor rhinitis, exaggerated nasal cycle, acute viral rhinitis, acute viral rhinosinusitis, acute bacterial rhinitis, acute bacterial rhinosinusitis, chronic inflammatory or infectious rhinosinusitis, rhinitis medicamentosa, sarcoidosis, Wegener's granulomatosis, Churg–Strauss syndrome, and rhinoscleroma [20].

In the present study, approximately 85.5% of the patients presented with DNS. Similar findings were previously reported in diverse studies. In a study that investigated the correlation of nasal septal deviations and chronic rhinosinusitis, DNS was found in 49 (81.7%) subjects; 11 (18.3%) had it in both right and left sides, 16 (26.7%) in the right side alone, and 22 (36.7%) in the left side. The commonest types of septal deviations in the left side were posteroinferior (10, 16.66%) and anteroinferior (7, 11.7%). In the right side, the corresponding numbers were 9 (15%) and 7 (11.7%) [21]. Some studies have reported nasal septal deviation in 20–31% of the community and also revealed that severe deviation predisposed the individuals to rhinosinusitis [22, 23].

Nasal septoplasty is one of the most common procedures in otolaryngology owing to the high prevalence of nasal obstruction from septal deviation [24]. As stressed in the literature, identification of the C- and S-shaped deformities at the time of planning remains crucial for identifying potentially complex surgeries compared to less technically challenging operative interventions such as septal tilts [25, 26].

*Conclusion*. Nasal obstruction is prevalent in Northern Saudi Arabia with peaks being in the years 2016 and 2014 with the most etiological factor being DNS. DNS is commonly seen among men in Northern Saudi Arabia and most patients underwent septoplasty in their twenties. Septoplasty has a good prospect to be accepted as a quick practice, with patient effective perioperative care being the basis of good outcomes.

## Figures and Tables

**Table 1:** Study subjects by demographical characteristics.

Variable	Category	Males	Females	Total
Year of Diagnosis				
	2012	4	1	5
	2013	4	1	5
	2014	16	10	26
	2015	23	4	27
	2016	38	9	47
	2017	14	7	21
	Total	99	32	131
Age groups				
	<20 years	16	11	27
	21–25	29	14	43
	26–30	26	2	28
	31–35	17	2	19
	36+	11	3	14
Occupations				
	Employees	23	1	24
	Teachers	16	2	18
	Students	29	16	45
	Solders	9	0	9
	Medicals	1	1	2
	others	21	12	33

**Table 2:** Distribution of the study subjects by clinical presentations.

Variable	No	Mild	Moderate	Severe	Total
Nasal congestions	22	17	30	62	131
Nasal blockages	8	15	26	82	131
Breathing troubles	17	17	27	70	131
Sleeping troubles	21	20	20	70	131
Exercise problems	38	31	28	34	131

**Table 3:** Distribution of the study subjects by gender and clinical presentations.

Variable	Category	No	Mild	Moderate	Severe	RR (95% CI)
Nasal congestions						
	Males	17	11	23	48	0.98 (0.8311 to 1.1736)
	Females	5	6	7	14	
Nasal blockages						
	Males	6	8	19	66	0.96 (0.2058 to 4.5683)
	Females	2	7	7	16	
Breathing troubles						
	Males	11	11	22	55	1.09 (0.9148 to 1.3116)
	Females	6	6	5	15	
Sleeping troubles						
	Males	15	13	15	56	1.04 (0.8670 to 1.2579)
	Females	6	7	5	14	
Exercise problems						
	Males	26	25	20	28	1.17 (0.8802 to 1.5815)
	Females	12	6	8	6	

**Table 4:** Distribution of the study subjects by age and clinical presentations.

Variable	Category	No	Mild	Moderate	Severe	Total
Nasal congestions						
	<20 years	2	8	5	12	27
	21–25	12	1	14	16	43
	26–30	5	3	3	17	28
	31–35	2	3	4	10	19
	36+	1	2	4	7	14
	Total	22	17	30	62	131
Nasal blockages						
	<20 years	2	0	9	16	27
	21–25	3	8	8	24	43
	26–30	1	3	4	2	28
	31–35	1	2	3	13	19
	36+	1	2	2	9	14
Breathing troubles						
	<20 years	5	3	6	13	27
	21–25	6	7	6	24	43
	26–30	4	2	4	18	28
	31–35	2	2	6	9	19
	36+	0	3	5	6	14
Sleeping troubles						
	<20 years	5	3	4	15	27
	21–25	8	9	7	19	43
	26–30	5	2	6	15	28
	31–35	3	4	3	9	19
	36+	0	2	0	12	14
Exercise problems						
	<20 years	12	6	5	4	27
	21–25	12	6	12	13	43
	26–30	6	9	6	7	28
	31–35	6	4	4	5	19
	36+	2	6	1	5	14

**Table 5:** Distribution of the study subjects by occupation and clinical presentation.

Variable	Category	No	Mild	Moderate	Severe	Total
Nasal congestions						
	Employees	3	2	8	11	24
	Teachers	1	1	4	12	18
	Students	9	8	9	19	45
	Solders	2	2	1	4	9
	Medicals	0	1	1	0	2
	Others	7	3	7	16	33
Nasal blockages						
	Employees	0	2	6	16	24
	Teachers	0	3	2	13	18
	Students	2	3	11	29	45
	Solders	1	1	2	5	9
	Medicals	0	1	1	0	2
	Others	5	5	4	19	33
Breathing troubles						
	Employees	2	3	6	13	24
	Teachers	1	1	4	12	18
	Students	5	5	10	25	45
	Solders	2	2	2	3	9
	Medicals	1	1	0	0	2
	Others	6	5	5	17	33
Sleeping troubles						
	Employees	2	1	5	16	24
	Teachers	1	4	2	11	18
	Students	8	6	7	25	45
	Solders	1	1	2	5	9
	Medicals	0	1	1	0	2
	Others	9	7	3	14	33
Exercise problems						
	Employees	5	6	5	8	24
	Teachers	5	2	5	5	18
	Students	17	9	8	11	45
	Solders	3	4	1	1	9
	Medicals	1	0	1	0	2
	Others	7	10	8	8	33
